# Thioredoxin Interacting Protein Is a Potential Regulator of Glucose and Energy Homeostasis in Endogenous Cushing's Syndrome

**DOI:** 10.1371/journal.pone.0064247

**Published:** 2013-05-17

**Authors:** Tove Lekva, Jens Bollerslev, Afaf Sahraoui, Hanne Scholz, Hege Bøyum, Johan Arild Evang, Kristin Godang, Pål Aukrust, Thor Ueland

**Affiliations:** 1 Section of Specialized Endocrinology, Department of Endocrinology, Oslo University Hospital, Oslo, Norway; 2 Research Institute of Internal Medicine, Oslo University Hospital, Oslo, Norway; 3 Section of Clinical Immunology and Infectious Diseases, Oslo University Hospital, Oslo, Norway; 4 Section for Transplantation, Oslo University Hospital, Oslo, Norway; 5 Institute for Surgical Research, Oslo University Hospital, Oslo, Norway; 6 National Resource Centre for Women's Health, Division of Obstetrics and Gynecology, Oslo University Hospital, Oslo, Norway; 7 Institute of Clinical Medicine, University of Oslo, Oslo, Norway; Medical University Innsbruck, Austria

## Abstract

Recent studies have described bone as an endocrine organ regulating glucose metabolism, with insulin signaling regulating osteocalcin secretion and osteocalcin regulating β cell function. We have previously demonstrated increased bone expression of TXNIP in patients with endogenous Cushing's syndrome (CS), and we hypothesized that TXNIP could contribute to the dysregulated glucose metabolism in CS. We studied 33 CS patients and 29 matched controls, with bone biopsies from nine patients, before and after surgical treatment. *In vitro*, the effect of silencing TXNIP (siTXNIP) in osteoblasts, including its effect on human islet cells, was examined. Our major findings were: (i) The high mRNA levels of TXNIP in bone from CS patients were significantly associated with high levels of glucose and insulin, increased insulin resistance, and decreased insulin sensitivity in these patients. (ii) Silencing TXNIP in osteoblasts enhanced their OC response to insulin and glucose and down-regulated interleukin (IL)-8 levels in these cells. (iii) Conditional media from siTXNIP-treated osteoblasts promoted insulin content and anti-inflammatory responses in human islet cells. We recently demonstrated that the thioredoxin/TXNIP axis may mediate some detrimental effects of glucocorticoid excess on bone tissue in CS. Here we show that alterations in this axis also may affect glucose metabolism in these patients.

## Introduction

Glucose intolerance and overt diabetes mellitus are classical parts of endogenous Cushing's syndrome (CS), and insulin resistance is a well known feature of cortisol excess [1]. CS and the metabolic syndrome share common characteristics including hyperglycemia, abdominal obesity, reduced HDL cholesterol and elevated triglyceride levels, and hypertension [2], [3]. In addition to its metabolic effects, cortisol excess also inhibits bone formation, increases bone resorption, impairs calcium absorption from the gut, and affects the secretion of several hormones, cytokines, and growth factors with potential influence on bone metabolism [1], and notably some of these mediators may also influence other metabolic pathways [4]

Recent studies have shown a reciprocal relationship between bone and energy metabolism, whereby imbalance in bone remodeling seems to be of importance for glucose metabolism and insulin resistance and *vice versa*, at least in experimental models [5], [6]. Mice lacking the osteocalcin (OC) gene had an abnormal amount of visceral fat, decreased β-cell proliferation, glucose intolerance, and insulin resistance [7]. Further, insulin signaling in osteoblasts was shown to enhance circulating undercarboxylated OC (ucOC) and impact glucose homeostasis [8], [9]. We and others have previously shown that CS patients have low levels of OC and display low bone mass [10]–[12]. While some clinical studies demonstrate associations between circulating OC or ucOC and glucose metabolism in different populations [13], [14], the association between bone and glucose metabolism in CS is unknown.

Thioredoxin 1 (TRX) is involved in the regulation of intracellular redox status and is induced and secreted in response to oxidative stress and inflammation. High circulating levels of TRX have been found in patients with diabetes mellitus, hypertension, hypercholesterolemia, atherosclerosis [15]–[17], and cancers [18]. A number of studies suggest that TRX activity can be modulated by the interaction with thioredoxin interacting protein 1 (TXNIP). TXNIP is a protein with a variety of functions and considered a negative regulator of TRX functions and/or expression [19]. In pancreatic islets cells, the expression of TXNIP has been shown to be increased by glucose and to promote apoptosis and potentially glucotoxicity and β-cell loss in insulin resistance and diabetes [20]. Silencing TXNIP expression in adipocytes and in skeletal muscle was found to enhance glucose uptake, and TXNIP was also elevated in muscles of prediabetics and diabetics [21]. Moreover, TXNIP deficient mice show hyperinsulinemia with marked hypoglycemia during starvation [22], [23], further linking TXNIP to β-cell function.

We have previously performed a microarray study on mRNA isolated from bone biopsies in CS patients to identify genes that could be involved in glucocorticoid induced osteoporosis. TXNIP was one of the most regulated genes and further investigation demonstrated that it is regulated by glucocorticoids in bone cells *in vitro*. Furthermore, we found that TXNIP expression in osteoblasts was a major regulator of OC production. Based on the pronounced insulin resistance observed in CS and low circulating OC levels, we hypothesized that long-term exposure to glucocorticoids could impact insulin resistance through TXNIP mediated mechanisms on OC in bone. This was investigated *in vivo* by looking at associations between TRX/TXNIP, OC and indices of insulin resistance and *in vitro* by modulating TXNIP in osteoblasts and investigating effects of these osteoblasts on pancreatic islets.

## Materials and Methods

### Subjects

Thirty-three patients diagnosed with CS were recruited based on clinical evaluation and biochemical workup as previously detailed [24]. In 9 of the patients, trans iliac crest bone biopsies with an inner diameter of 6 mm were obtained under local anesthesia and stored in liquid nitrogen until analysis at baseline, and then again 3.0±1.3 (mean ± SD) months after transsphenoidal surgery of a corticotroph pituitary adenoma. A group of 29 healthy subjects matched by age, sex and BMI were used as controls. All blood samples were drawn after an overnight fast in the morning (8 am). All patients and controls gave signed informed consent, and the study was approved by the Regional Committee of Medical and Health Research Ethics in Eastern Norway and conducted according to the Declaration of Helsinki II.

### Biochemical markers

Undercarboxylated OC was measured by enzyme immunoassays (EIA) provided by Takara (Shiga, Japan) and insulin by radioimmunoassay (RIA) (Diagnostic Products Corp., Los Angeles, CA). TRX and cortisol was measured as previously reported [24], [25]. Interleukin 8 (IL-8) in supernatants was measured by EIA from R&D Systems (Minneapolis, MN), homeostasis model assessment index of IR (HOMA-IR) and quantitative insulin sensitivity check index (QUICKI) were estimated based on fasting glucose and insulin [26], [27].

### RNA isolation from bone biopsies and cultured cells

Extraction of total RNA was performed using Trizol (Invitrogen, Carlsbad, CA) as previously described [24]. RNA was purified using QIAGEN RNeasy micro kit with dnase treatment (Qiagen, Valencia, CA). The integrity was assessed using Agilent 2100 Bioanalyzer (Agilent Technologies, Santa Clara, CA), and concentrations determined by OD readings on Nanodrop ND-1000 Spectrophotometer (Nanodrop Technologies,Wilmington, DE).

### Microarray

Microarray analysis was performed as previously described [24], using biotin-labeled cRNA probes hybridized to HG U133 Plus 2.0 Arrays (Affymetrix, Inc., Santa Clara, CA). All microarray raw data has been deposited on Gene Expression Omnibus (GEO) under accession number GSE30159.

### Real-time RT-PCR

Reverse transcription was performed using a High Capacity cDNA Archive Kit (Applied Biosystems, Foster City, CA). Quantification of mRNA was performed using standard curve method of the ABI Prism 7500 (Applied Biosystems). For real-time RT-PCR, sequence specific exon-exon spanning oligonucleotide primers were designed using Primer Express software version 2.0 (Applied Biosystems) and can be supplied on request.

### Western blot

Extraction of protein from the bone biopsies was performed using the Trizol method (Invitrogen, Carlsbad, CA) after RNA and DNA was removed. The protein pellet was dissolved in 1% SDS and stored in −70 C. Eighty micrograms of total protein of each biopsy were incubated at 97°C for 2 minutes before separated on Any kD SDS-polyacrylamide gels (Bio-Rad Laboratories, Hercules, CA) and transferred onto polyvinylidene difluoride membranes (NEN™, Life Science, Boston, MA). Membranes were incubated with mouse anti-human TXNIP (MBL International, Woburn, MA) antibody over night at 4°C and 1 hour at room temperature with anti-mouse IgG (Cell Signaling Technology, Denver, MA) horseradish peroxidase (HRP) -conjugated secondary antibody. HRP signals were developed using the ECL Plus Western blotting detection system (GE Healthcare Bio-Sciences, Uppsala, Sweden) and visualized by Fujifilm Luminiscent Image Analyzer 4000 mini. To ensure equal loading of protein, the blots were stained with Ponceau S solution (Sigma-Aldrich, St Louis, MO).

### Silencing of TXNIP gene expression in hFOB

A pool of four siRNA duplexes specific for human TXNIP (siTXNIP) and scramble control pool (siSCR) were designed by Dharmacon (Lafayette, CO), and experiments preformed as previously reported [25]. The sense strand sequences were, TXNIP1: 5′-GAAUACAUGUUCCCGAAUUUU-3′, TXNIP2: 5′-GCAAACAGACUUCGGAGUAUU3′, TXNIP3:5′-CGAACCAGCUCUGAGAUGAUU-3′, TXNIP4: 5′GUCAGAGGCAAUCAUA UUAUU-3′. hFOB cells (human fetal osteoblast cell line 1.19 American Type Culture Collection, CRL-11372, Rockville, MD) were grown and transfected in twelve-well plates (5×10^4^ cells/well) for 24 hours at 33.5°C (40% confluent) in antibiotic-free medium containing 10% FCS. For transfection, 50 nM siRNA duplexes and 6 µl HiPerFect transfection reagent (Qiagen, Hilden, Germany) were prepared in OptiMem with glutamax-1 (Gibco-Invitrogen) at a final volume of 300 µl in 12-well plates (Costar, Cambridge, MA). After 4 hours, 300 µl medium with 0.6% Ultrozer (Pall Corporation, Port Washington, NY) was added to the cells for the remaining period of 24 hours at 33.5°C, and the temperature was shifted to 39.5°C for 48 hours to induce differentiation. Finally, cells were treated with glucose (5.6 and 50 mM), insulin (2.5 and 100 nM) and vehicle in quadruplicate cultures for different times, as indicated. In different experiments media from the silenced TXNIP osteoblasts were used to stimulate islet cells. The media was prepared as the vehicle above for the time point of 24 and 48 hours. A combination of the supernatant of these two time points was used for treatment experiments on the islets. The glucose level in the supernatants was measured in different batches of supernatant with Accu-chek compact strips (Roche, Werk Penzberg, Germany).

### Human pancreatic islets treated with siTXNIP osteoblast conditioned media

Following obtained consent from the organ donor registry or relatives, pancreata from deceased donors were transported from the donor hospital to the laboratory for islet isolation in Uppsala, Sweden. Islets were isolated according to the automated method, refined by the Nordic Network for Islet Transplantation [28]. Within 2–5 days from isolation aliquots of purified islet preparations from six different donors were placed into 90 mm Petri dishes and cultured with media from hFOB (siTXNIP- and siSCR-treated condition media) for 24 hours at 37°C in a 5% CO2 incubator. Twenty equally distributed islets were handpicked and homogenized before acid-ethanol extraction overnight at 4°C. The intracellular insulin content was measured in homogenized islets by human Insulin EIA (Mercodia, Uppsala, Sweden) and normalized to DNA content determined by Quant-iT picogreen dsDNA assay kit (Invitrogen). Cell pellets (mRNA analyses) were harvested and stored at −80°C until analysed as described above.

### Statistical analysis

Unless stated otherwise, all values are expressed as mean±SEM. In vivo data were analyzed by non-parametric statistics (Wilcoxon ranked sum, Mann-Whitney U, Spearman correlation) while in vitro data were analyzed by parametric statistics (for analysis of time course and dose response, one way ANOVA and unpaired T-tests were used). A p-value of *p*<0.05 was considered significant. All *in vitro* studies were performed three times with representative experiments presented.

## Results

Comparing patients (n = 33) *vs.* controls (n = 29) there were no significant differences in age (median [25^th^, 75^th^ percentile]: 41 [31, 50] *vs.* 42 [38, 51] yrs., p = 0.23), gender (25 *vs.* 19 women, p = 0.38 or BMI (30.1 [25.4, 33.9] *vs.* 27.1 [24.2, 31.2] kg/m^2^, p = 0.17). As expected, patients with CS were characterized by markedly elevated cortisol levels (585 [478, 764] *vs.*379 [293, 491] nmol/L, p<0.001).

### Associations between the TRX/TXNIP axis and glucose metabolism in vivo

**Figure 1:**
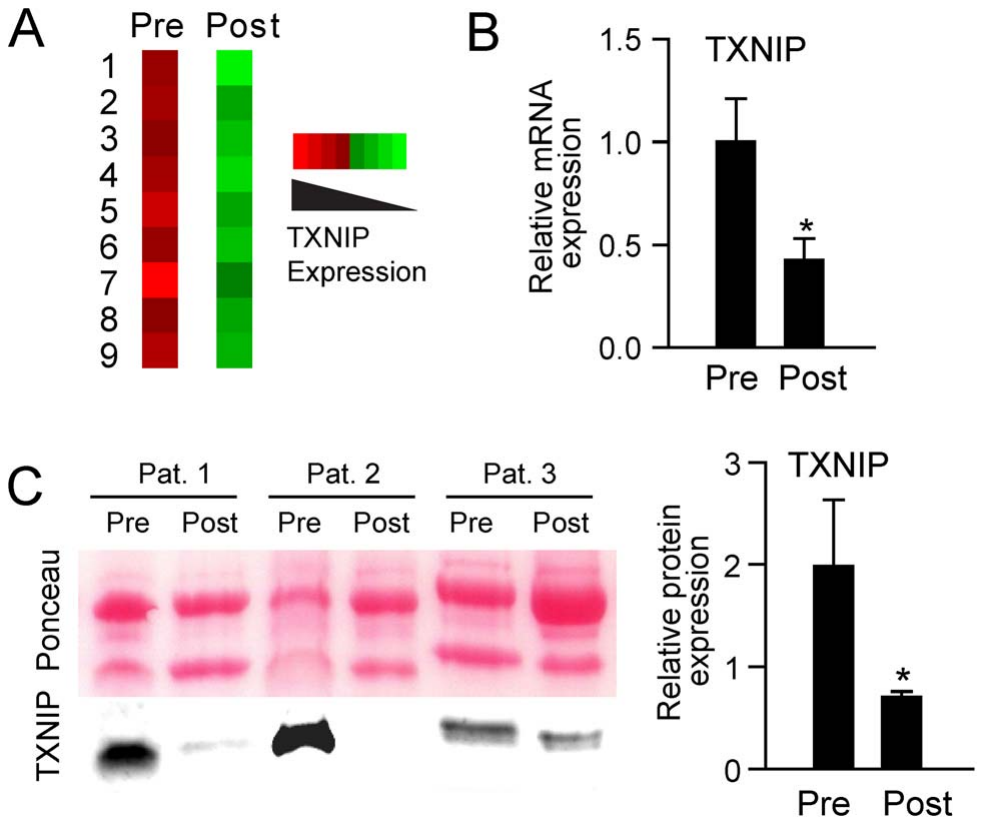
TXNIP expression in nine paired bone biopsies pre and 3 months post surgery. (A) microarray analysis. B) mRNA expression normalized to β-actin and expressed as relative mRNA levels. (C) protein levels in three paired bone biopsies pre and 3 months post surgery and Ponceau S total protein staining by western blot and (D) protein levels in all the nine paired bone biopsies related to Ponceau S total protein staining.

**Figure 2:**
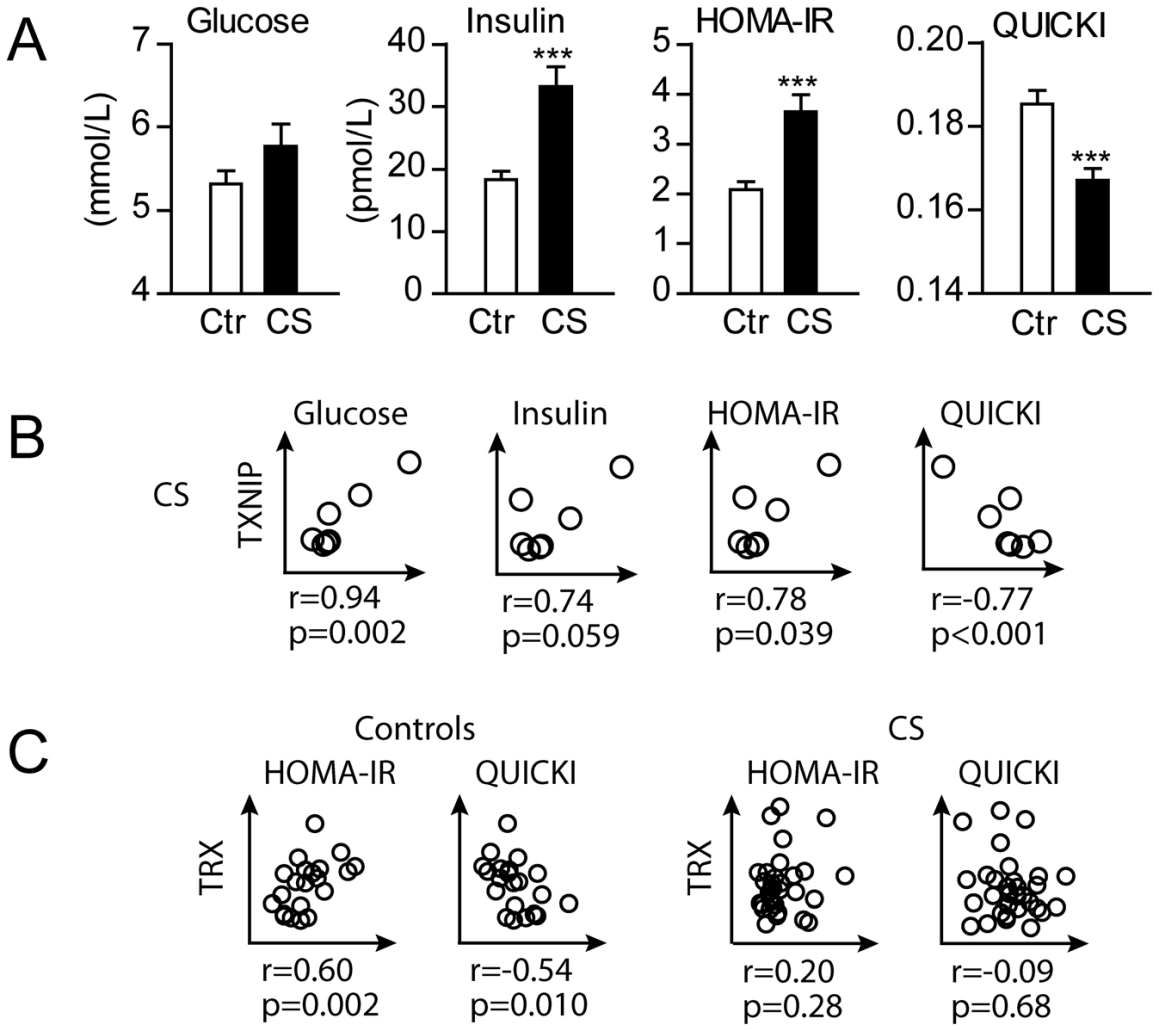
Associations between the TRX/TXNIP axis and glucose metabolism *in vivo*. (A) Fasting glucose and insulin levels and indices of insulin resistance (HOMA-IR) and insulin sensitivity (QUICKI) in 33 CS patients with active disease and 29 age-, sex- and BMI-matched controls. (B) Correlation between TXNIP mRNA expression in iliac crest bone biopsies from 7 CS patients with active disease and measures of glucose metabolism (due to shortage of serum from two of the patients). (C) Correlation between serum TRX levels and insulin resistance and insulin sensitivity in controls and CS patients with active disease.

We have previously demonstrated markedly increased TXNIP mRNA levels in iliac crest biopsies in CS patients with active disease, significantly correlated to high plasma levels of TRX, with decreased mRNA levels of TXNIP post-treatment [25]. Figure 1A–B shows the microarray and mRNA data of TXNIP pre and median 3 months after transsphenoidal surgery of a corticotroph pituitary adenoma in nine paired bone biopsies [25], while Figure 1C–D show a similar pattern at the protein level with a decreased TXNIP following surgery. When examining glucose metabolism in these patients, we found no differences in fasting glucose levels between CS and controls, but the patients were characterized by markedly elevated fasting insulin levels (Figure 2A) resulting in an increased insulin resistance and decreased insulin sensitivity as assessed by HOMA and QUICKI calculators, respectively (Figure 2A). Further, when looking at associations with TXNIP mRNA expression in bone, although limited by few observations, positive correlations were seen between TXNIP and fasting glucose, insulin and HOMA-IR, while QUICKI was negatively correlated with TXNIP (Figure 2C). Finally, while circulating TRX was correlated with insulin (r = 0.54, p = 0.008), insulin resistance (i.e. HOMA-IR, Figure 2C) and decreased insulin sensitivity (i.e. QUICKI, Figure 2C) in controls, no associations with indices of glucose metabolism were found in CS patients (Figure 2C), potentially suggesting a dysregulated TRX/TXNIP axis in these patients.

### Associations between osteocalcin and glucose metabolism in vivo

**Figure 3:**
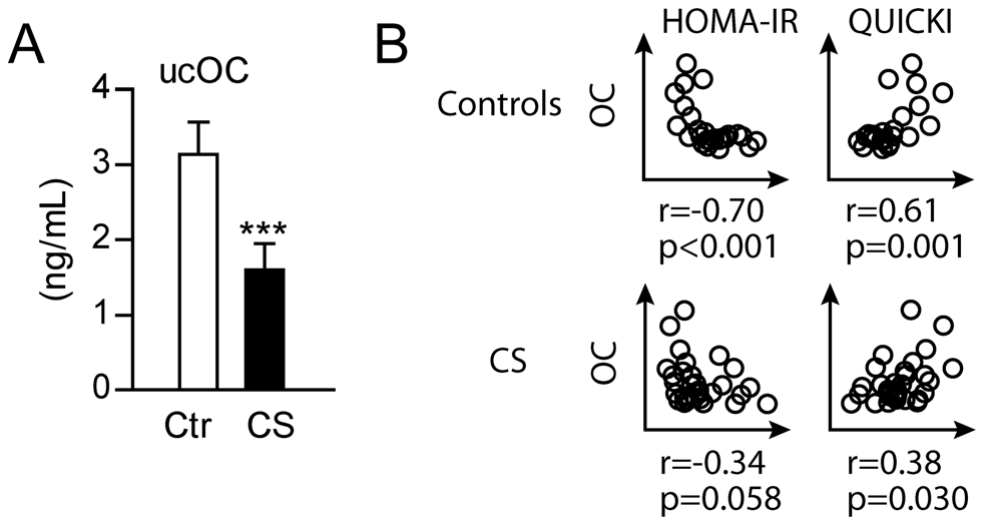
Associations between OC and glucose metabolism *in vivo*. (A) Serum levels of ucOC in 33 CS patients with active disease and 29 age-, sex- and BMI-matched controls. (B) Correlation between serum levels of OC and indices of insulin resistance (HOMA-IR) and insulin sensitivity (QUICKI) in controls and CS patients with active disease.

Insulin signaling in osteoblasts has recently been shown to influence glucose metabolism by regulating circulating ucOC [9]. Figure 3A shows that patients with CS have markedly reduced serum levels of ucOC compared to controls. However, when correlating ucOC with indices of glucose metabolism no significant associations were demonstrated (data not shown). In contrast, serum levels of the intact OC (1–72) carboxylated molecule, that previously have been measured in this population [25], were negatively correlated with insulin resistance (i.e HOMA-IR) and positively correlated with sensitivity (i.e. QUICKI) in both patients and controls, with particularly strong associations among healthy controls (Figure 3B).

### Silencing of TXNIP in osteoblasts influences the glucose-dependent regulation of OC and the inflammatory response in these cells in vitro

**Figure 4:**
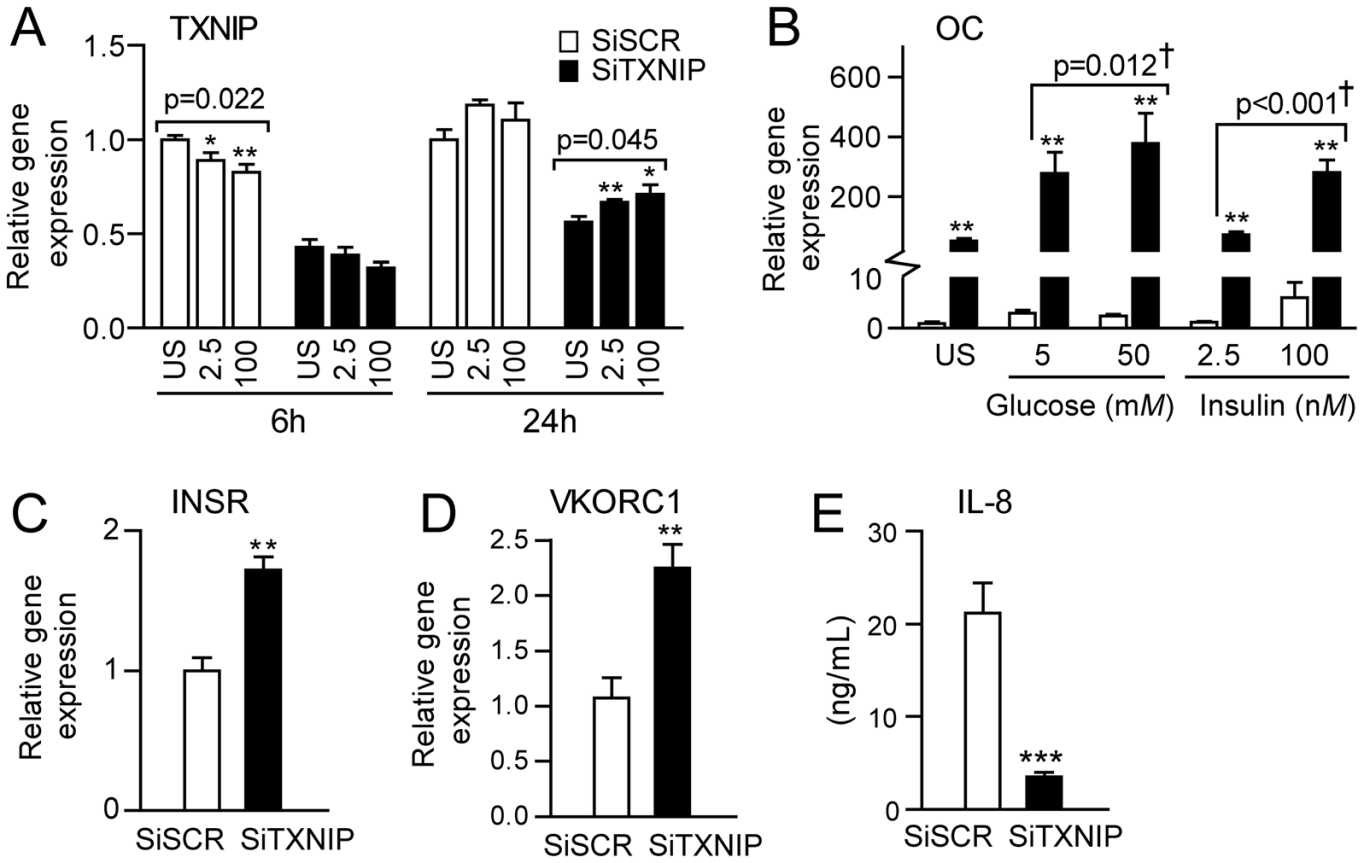
Silencing of TXNIP in hFOB. Gene expression of TXNIP (A) and OC (B) following 72 h silencing (24 h 33.5°C+48 h 39.5°C) using 25 nM siTXNIP (black columns) and 25 nM scrambled siSCR as control (white columns) in quadruplicate cultures before harvest. Cells were either treated with vehicle (US), glucose (5–25 mM) or insulin (2.5–100 nM), for 6 and 24 hours (TXNIP) and 24 hours (OC), in quadruplicate cultures before harvest. Gene expression of (C) INSR and (D) VKORC1 following 72 h silencing as described in A. mRNAs for TXNIP, OC, INSR and, VKORC1 were normalized to β-actin and expressed as relative mRNA levels. P-values represent one way ANOVA. (E) Protein levels of IL-8 in the supernatant following 72 h silencing as described in A. Results are presented as mean ± SEM; * *p*<0.05, ** *p*<0.01 and *p*<0.001*** indicate difference between siSCR and siTXNIP treated cells.

We have previously shown that silencing TXNIP in the osteoblast cell line hFOB for 2 days reduced mRNA expression (>50%) and diminished protein expression compared to scrambled treated controls, and that silencing TXNIP mRNA in osteoblasts has a strong effect on the regulation of OC in these cells (∼50-fold upregulation) [25]. In the present study we examined if the effect of TXNIP and OC was dependent on glucose and insulin. After silencing of TXNIP, we treated hFOB with different doses of glucose and insulin, and found TXNIP mRNA expression to be significantly decreased after 6 hours by insulin in scramble treated cells, and significantly increased by insulin after 24 hours in TXNIP silenced cells (Figure 4A), although similar trends were observed regardless of silencing. No significant effects were observed for glucose (data not shown). After 24 hours culture, we found OC mRNA to be dose-dependently increased after addition of glucose and insulin (Figure 4B), but notably, only in cells with reduced TXNIP expression (i.e. TXNIP siRNA). Moreover, silencing TXNIP, without any addition of glucose and insulin, markedly increased the mRNA levels of the insulin receptor (INSR), vitamin K-epoxide reductase complex subunit 1 (VKORC1), and markedly decreased the levels of IL-8 secretion, a prototypic inflammatory chemokine, in these cells as compared to scramble control (Figure 4C–E).

### Human islets treated with condition media from silenced TXNIP osteoblasts shows increased function

**Figure 5:**
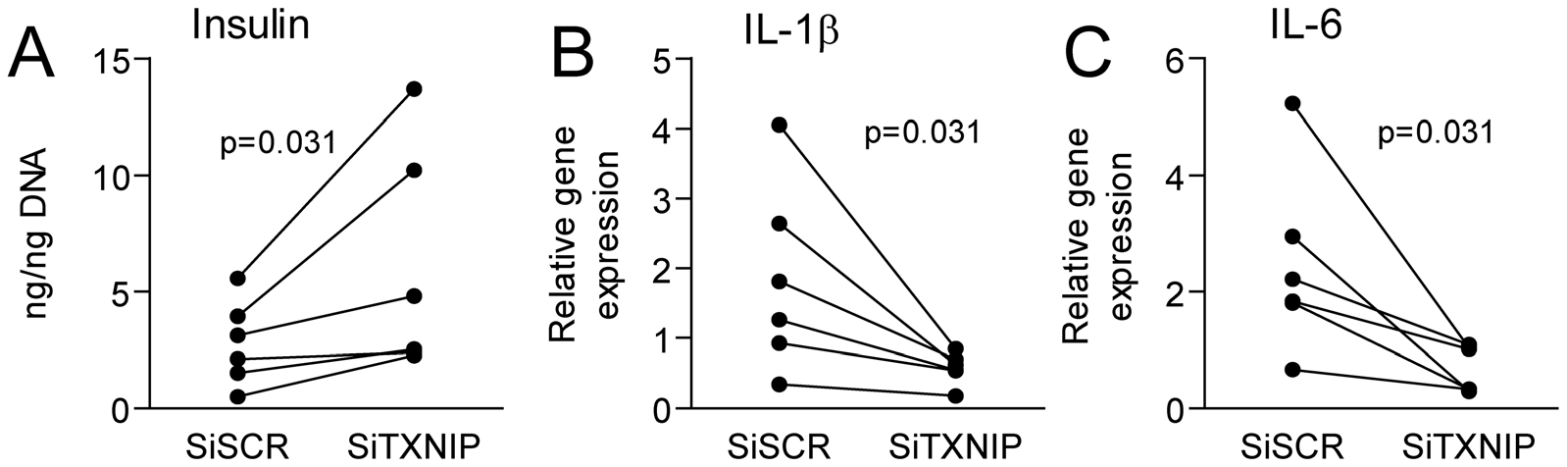
Human islets treated with silenced TXNIP OB condition media showed increased function. Islets were treated with siTXNIP and siSCR OB medium for 24 h. (A) Insulin content normalized to DNA in cell extracts from six different donors (1–2 cultures per donor). (B–C) Gene expression for IL-1β and IL6 normalized to β-actin and expressed as relative mRNA levels.

Our findings so far suggest that TXNIP may modulate osteoblast expression of factors that may have impact on human pancreatic islet function (i.e. OC and chemokines). To further elucidate these issues we stimulated human islets with conditioned media from hFOB treated with siTXNIP or siSCR control. As depicted in Figure 5, conditional media from siTXNIP treated osteoblasts induced increased insulin content and decreased mRNA expression of IL-1β and IL-6, representing inflammatory cytokines with potential harmful effect on islet cells, as compared to conditional media from siSCR treated osteoblasts. These findings may suggest beneficial effects of decreased TXNIP levels in osteoblasts on the functional capacity of human islets. However, somewhat surprisingly, the glucose level was increased in media from silenced TXNIP cells compared to silenced scramble control cells (n = 4, mean± SEM; 15.8±6.2 vs. 10.2±6.4 mM, p = 0.05 respectively), which could have contributed to the different effects of these supernatants.

## Discussion

We recently demonstrated that the TRX/TXNIP axis may mediate some detrimental effects of glucocorticoid excess on bone tissue in CS. Here we show that alterations in this axis also may affect glucose metabolism in these patients. Thus, in the present study we show (i) an association between high TXNIP mRNA expression in bone and high levels of glucose and insulin, increased insulin resistance, and decreased insulin sensitivity in CS patients; (ii) that decreased TXNIP levels in osteoblasts enhanced their OC response to insulin and glucose and down-regulated IL-8 levels in these cells; and that (iii) conditional media from siTXNIP-treated osteoblasts promoted insulin content and anti-inflammatory responses in human islets cells.

TXNIP has been shown to modulate overall gene transcription and enhance β-cell death resulting in impaired insulin secretion [29], [30]. We have recently showed increased mRNA levels of TXNIP in bone from CS patients [25]. Herein we show a strong correlation between increased bone expression of TXNIP and indices of disturbed glucose metabolism in CS patients. Insulin is reported to down-regulate TXNIP in skeletal muscle and adipose tissue [21], and skeletal muscle is yet considered a stronger determinant of insulin sensitivity than bone, but unfortunately we did not have access to muscle biopsies from CS patients. However, in hFOB the TXNIP was downregulated during short-term culture (i.e. 6 hours), but in contrast, cultures stimulated for 24 hours with insulin, which may be more relevant for the *in vivo* situation in CS patients, displayed increased levels of TXNIP. The positive association between TXNIP from bone and insulin levels is in contrast to the reciprocal relationship in healthy individuals reported by Parikh et al [21] and the interaction between TXNIP and insulin may thus differ between these groups of individuals as well as tissues investigated (i.e. bone, skeletal muscle and adipose tissue). Recent work has suggested a relationship between bone and energy metabolism [6]. Although our clinical data do not necessarily reveal any causal relationship, it is tempting to hypothesize that such interactions also may be operating in CS patients, mediating harmful effects on glucose metabolism. TRX attenuates the effects of TXNIP, and our previous report of increased serum levels of TRX in CS patients may apparently seem in conflict with our present data. However elevated circulating levels of TRX, as observed in metabolic disorders and type 2 diabetes mellitus, have been suggested to reflect a compensatory response to protect against the detrimental effects of TXNIP in an attempt to maintain β-cell function [15], [16], [31], and such mechanisms could also be operating in untreated CS. On the other hand, the lack of association between TRX and parameters of glucose metabolism in CS patents, but not in healthy controls, could suggest that a compensatory increase in TRX may not be adequate to limit enhanced TXNIP activity in CS patients.

Recently, OC has been suggested as a metabolic hormone regulating glucose homeostasis in experimental models [32]. Yet, confirmatory *in vivo* data in different patient populations characterized by perturbed glucose metabolism are limited, at least in relation to glucocorticoid excess. In mice lacking the INSR in osteoblasts, a decrease in both total OC and ucOC was observed in serum, leading to decreased bone formation and development of peripheral adiposity and insulin resistance [9]. Such mechanisms could be operating *in vivo* in CS as well. Based on the low OC, and in particular ucOC, in these patients, the potential beneficial effects of OC on β-cell function and insulin secretion could be reduced. Indeed, within healthy controls, high circulating OC was associated with markers of lower insulin resistance and increased insulin sensitivity. Although not as augmented, these associations were also present in CS, supporting that decreased OC could mediate some adverse effects of enhanced TXNIP expression in bone on insulin signaling and glucose metabolism in these patients. However, a local elevation of TXNIP in other tissues could also have direct negative effects on glucose metabolism in these patients. Indeed, hyperglycemia has been shown to increase TXNIP in adipocytes, and enhance inflammation in adipose tissue [33], which in turn may adversely affect glucose metabolism.

We recently reported that silencing TXNIP expression in human osteoblasts markedly increases OC expression and secretion in these cells [25]. In the present study we show that this effect of down-regulated TXNIP was further enhanced by glucose and insulin. Thus, the increased OC expression found when silencing TXNIP was amplified with higher concentrations of glucose and insulin. *In vivo*, this could indicate that a beneficial increase in OC in response to elevated blood glucose and/or insulin levels may be reduced in the presence of high TXNIP expression. Although glucocorticoids directly may modulate OC expression in osteoblasts by repressing activity of the OC gene promoter [34], our data suggest that enhanced TXNIP expression in bone also may contribute to depressed OC levels in CS and indirectly influence glucose metabolism. The mechanisms of the suppressive effect of TXNIP on OC synthesis and secretion is not known, but we previously found that silencing TXNIP increased typical osteoblast markers and indicate that it is important in regulating differentiation of osteoblasts, and that it also has suppressive effects by regulating caspase [25]. Further, we have data suggesting that TXNIP may regulate the carboxylation status of OC. We found that vitamin K-epoxide reductase complex (VKORC1) mRNA, the gene for the protein being the rate limiting step in OC carboxylation, increased following silencing of the TXNIP gene in osteoblasts, thereby increasing the capacity of post-transcriptional γ-carboxylation. Although the release and source of the different OC forms to the circulation is not fully elucidated, it seems that γ-carboxylation is important for OC affinity for calcium and hydroxyapatite. Thus, a decreased VKORC1 in the presence of increased TXNIP would potentially lead to decreased OC bound to bone matrix and thus less undercarboxylated OC released during bone resorption. On the other hand, a decrease in TXNIP and increase in both VKORC1 and OC following successful treatment of CS would increase γ-carboxylation and binding of OC to bone matrix and subsequent release during bone resorption. Thus, the attenuating effect of TXNIP on OC may reflect a general suppressive effect on osteoblast differentiation although we cannot exclude a direct regulation of OC transcription. In addition, some post-translational effects associated with the carboxylation status of OC may be present *in vivo*. Our finding that silencing TXNIP in osteoblasts was associated with a twofold increased INSR expression further supports that TXNIP can influence insulin signaling in these cells. Interestingly, the phenotype of transgenic mice lacking the INSR in osteoblasts, with decreased circulating OC, decreased bone formation and insulin resistance [9], are similar to the clinical features characterizing CS patients [35], [36].

TXNIP has been shown to modulate inflammation and oxidative stress in β-cells, including IL-1β which is known to be a key mediator of islet dysfunction and destruction in diabetes [37]. Our finding in the present study, that conditioned media from osteoblasts with low TXNIP expression increases islet function as demonstrated by enhanced insulin content and decreased production of the inflammatory cytokines IL-1β and IL-6, supports the concept that bone turnover and activity may influence global energy metabolism, at least partly through TXNIP-related mechanisms. Our study does not specifically identify which TXNIP regulated mediators that are involved in modulating β-cell function, and several candidates produced by osteoblasts (e.g. inflammatory mediators) could potentially be involved in addition to OC. Studies on the mechanisms behind these results are lacking and is a limitation to the present study. We found glucose to be increased in the siTXNIP osteoblast media compared to siSCR osteoblast media, which clearly could have contributed to the increased insulin content in islets stimulated with this media and limiting the significance of this finding. Moreover, and with clear relevance to our present study, a recent experimental paper demonstrated that the adverse effects of high-dose glucocorticoids on glucose handling in insulin target tissues are mediated, at least in part, through OC production in the skeleton [38]. They observed that osteoblast-targeted disruption of glucocorticoid signaling significantly attenuated the suppression of OC synthesis and prevented the development of insulin resistance and glucose intolerance. These new data together with our own data showing a pronounced effect of TXNIP silencing on OC expression as well as our *in vivo* data in CS patients may suggest that TXNIP could mediate some adverse effects of glucocorticoids on glucose metabolism through its influence on OC in osteoblasts. We have previously reported that patients with CS are characterized by elevated circulating levels of IL-8, inversely correlated with bone mass and serum OC levels [39]. IL-8 has been reported to have adverse effects on β-cell function [40]. Our observations that osteoblasts produce markedly more of this chemokine in the presence of TXNIP could suggest that IL-8 may be involved in the harmful interaction between osteoblasts and glucose metabolism in CS patients. However, these mechanisms will have to be further clarified in forthcoming studies.

Although relatively few patients were examined, our study indicates that a dysregulated TRX/TXNIP axis could mediate some of the detrimental effects of glucocorticoid excess on glucose homeostasis in CS patients, and suggests that skeletal tissue may be a key regulator of energy metabolism in these patients.
